# Axonal Regeneration and Neuronal Function Are Preserved in Motor Neurons Lacking ß-Actin *In Vivo*

**DOI:** 10.1371/journal.pone.0017768

**Published:** 2011-03-22

**Authors:** Thomas R. Cheever, Emily A. Olson, James M. Ervasti

**Affiliations:** Department of Biochemistry, Molecular Biology and Biophysics, University of Minnesota, Minneapolis, Minnesota, United States of America; Yale University, United States of America

## Abstract

The proper localization of ß-actin mRNA and protein is essential for growth cone guidance and axon elongation in cultured neurons. In addition, decreased levels of ß-actin mRNA and protein have been identified in the growth cones of motor neurons cultured from a mouse model of Spinal Muscular Atrophy (SMA), suggesting that ß-actin loss-of-function at growth cones or pre-synaptic nerve terminals could contribute to the pathogenesis of this disease. However, the role of ß-actin in motor neurons *in vivo* and its potential relevance to disease has yet to be examined. We therefore generated motor neuron specific ß-actin knock-out mice (*Actb*-MNsKO) to investigate the function of ß-actin in motor neurons *in vivo*. Surprisingly, ß-actin was not required for motor neuron viability or neuromuscular junction maintenance. Skeletal muscle from *Actb*-MNsKO mice showed no histological indication of denervation and did not significantly differ from controls in several measurements of physiologic function. Finally, motor axon regeneration was unimpaired in *Actb*-MNsKO mice, suggesting that ß-actin is not required for motor neuron function or regeneration *in vivo*.

## Introduction

The cytoskeletal protein actin has well characterized roles in many aspects of neuronal development and function from growth cone dynamics to the remodeling of dendritic spines [1], [2]. Although neurons of higher vertebrates express two nearly identical actin isoforms, ß- and γ-actin [3], ß-actin is thought to be the primary mediator of neuronal actin dynamics based on its unique post-transcriptional regulation. In cultured neurons, ß-actin is enriched at the leading edge of growth cones via an mRNA localization and local translation mechanism [4], [5]. The 3′-UTR of ß-actin mRNA contains a 54 nucleotide sequence called the zipcode which is bound co-transcriptionally by zipcode binding protein 1 (ZBP1, also known as IMP1 in humans, mIMP or CRD-BP in mice) [5]–[8]. ZBP1 facilitates the transport of ß-actin mRNA from the cell body to the growth cone while also inhibiting its translation [5]. Attractive guidance cues received by the growth cone initiate a signaling cascade that results in the release and translation of ß-actin mRNA, thereby generating a localized increase of newly translated ß-actin which is hypothesized to be an underlying mechanism behind growth cone turning [9], [10].

Although extensively characterized in cell culture models, less is known regarding the significance of ß-actin mRNA and protein localization in mammalian neurons *in vivo*. The most compelling evidence comes from a mouse model of Spinal Muscular Atrophy (SMA), a genetic disorder resulting from the selective loss of lower motor neurons [11]. Rossol and colleagues [12] observed a dramatic decrease in ß-actin mRNA and protein in the growth cones of cultured motor neurons from a mouse model of SMA, which correlated with a decrease in axonal elongation, growth cone size, and mislocalization of voltage gated calcium channels [13]. Interestingly, survival motor neuron (SMN), the protein depleted in SMA, interacts with two proteins known to bind the zipcode sequence of ß-actin mRNA: hnRNP-R and KSRP, the human homologue of a ZBP family member [12], [14]–[16]. These observations led to the hypothesis that reduced levels of ß-actin at growth cones in SMN-deficient motor neurons may cause axon guidance or nerve terminal defects ultimately resulting in motor neuron death.

A number of recent studies however have now shown motor neuron axon guidance and elongation is unperturbed in SMA mouse models, suggesting that decreased levels of ß-actin in motor neuron growth cones likely does not hinder axon guidance or elongation [17]–[19]. Yet, this data does not rule out whether ß-actin may play a role later in the development or maintenance of the neuromuscular junctions (NMJ), the specialized synapse between motor neurons and skeletal muscle. Given that previous studies have shown a mislocalization of voltage gated calcium channels in SMA motor neurons that correlated with the decrease of ß-actin in growth cones [13], one intriguing hypothesis is that ß-actin plays a crucial role in pre-synaptic NMJ structure and function, a potential pathogenic mechanism more in line with current *in vivo* findings from SMA mouse models [17], [18], [20]–[22].

We therefore set out to determine the function of ß-actin in mature motor neurons by conditionally ablating its expression with a floxed ß-actin mouse line [23] and the motor neuron specific Cre line, Mnx1-Cre [24] (see additional references in materials and methods). Surprisingly, motor neuron specific ß-actin knock-out (*Actb*-MNsKO) mice exhibited no overt phenotype, with motor neuron viability, NMJ, and muscle performance all preserved in comparison to controls. Peripheral nerve axonal regeneration was also unimpaired in *Actb*-MNsKO mice, indicating that ß-actin is dispensable for motor neuron function and regeneration *in vivo*.

## Results

### Generation of a motor neuron specific ß-actin knock-out mouse model (*Actb*-MNsKO)

**Figure 1:**
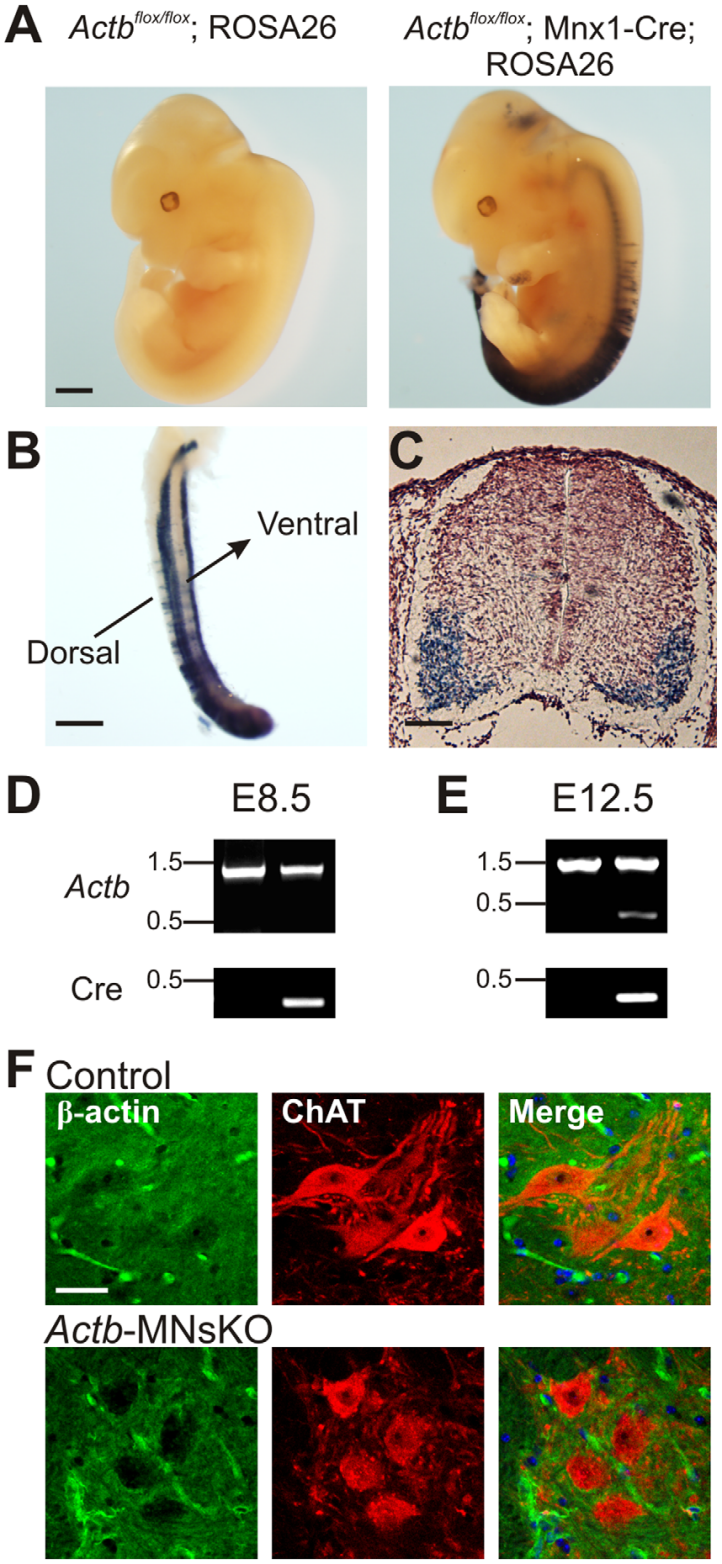
Verification of ß-actin ablation in motor neuron specific ß-actin knock-out mice. (**A**) *Actb ^flox/flox^* and *Actb ^flox/flox^*; Mnx1-Cre (*Actb*-MNsKO) mice were crossed to the ROSA26 reporter strain and stained for ß-galactosidase activity at E12.5. No background ß-galactosidase activity was noted in Mnx1-Cre (−) controls. In Mnx1-Cre (+) embryos, ß-galactosidase activity was present throughout the entire length of the spinal cord as seen in whole embryos (**A**) and isolated spinal cord (**B**). Scale bar 1 mm. (**C**) Stained cross sections through the spinal cord showed ß-galactosidase activity throughout the ventral horns confirming that Mnx1-Cre is expressed selectively in motor neurons in the developing spinal cord. Sections were counterstained with 0.1% neutral red. Scale bar 25 µm. (**D–E**) PCR genotyping of spinal cord DNA extracts from control and *Actb*-MNsKO embryos. One day prior to Mnx1-Cre expression at E8.5, no recombination product is detected in embryos with the Cre transgene (**D**). Following Cre expression and recombination at E12.5, the recombined *Actb* allele is present only in spinal cord DNA extracts from embryos harboring the Cre transgene and not in control embryos (**E**). (**F**) Twenty micron cryosections from the ventral horn of the lumbar enlargement of 6 month old control and *Actb*-MNsKO mice stained with antibodies against ß-actin and choline acetyltransferase (ChAT) to label motor neurons. Merged image also includes DAPI to label nuclei. Scale bar 30 µm.

In order to circumvent the early embryonic lethality observed in previous ß-actin knock-out mouse models [25], [26], floxed ß-actin mice [23] were crossed to the Mnx1-Cre transgenic mouse line to specifically ablate ß-actin in motor neurons. The floxed *Actb* line has been used to successfully ablate ß-actin from hair cells of the inner ear [23] while the Mnx1-Cre line has been used extensively to generate a number of motor neuron specific knock-out mouse lines [27]–[32]. We first confirmed the spatial and temporal expression of Mnx1-Cre by crossing *Actb*-MNsKO mice with a ROSA26 reporter line containing the ß-galactosidase gene with a floxed stop codon cassette. Thus, ß-galactosidase activity is present only in tissues where Cre recombinase is expressed and functional. At embryonic day 12.5 (E12.5), Cre recombinase activity was present throughout the ventral horns along the entire length of the developing spinal cord, confirming robust expression of Mnx1-Cre in motor neurons in *Actb*-MNsKO embryos (Figure 1A–C). Successful recombination of the *Actb* locus was confirmed by PCR of genomic DNA from embryonic spinal cord tissue at E12.5 (Figure 1E). Furthermore, spinal cord sections from control and *Actb*-MNsKO mice were stained for ß-actin protein and choline acetyltransferase (ChAT), a marker for motor neurons. ß-actin expression was ubiquitous throughout the spinal cord, although it was relatively weak in the cell bodies of control motor neurons (Figure 1F top), consistent with previous reports indicating that ß-actin is present in neuronal cell bodies only at low levels *in vivo* [33], [34]. However, no detectable ß-actin signal was present in motor neuron cell bodies from *Actb*-MNsKO mice, confirming motor neuron specific ablation of ß-actin (Figure 1F bottom). Finally, no detectable ß-actin signal was present in motor neuron cell bodies from a second line of ß-actin knock-out mice generated with the Nestin-Cre transgenic line (Figure S1A), which expresses Cre in motor neurons and other cells of the central nervous system (CNS-*Actb*KO) [35]–[37]. Thus, we have verified selective and robust motor neuron specific Cre expression by the Mnx1-Cre transgenic mouse line, recombination of the floxed *Actb* locus, and loss of ß-actin signal from motor neuron cell bodies in two independent lines by immunofluorescence, collectively establishing conditional ablation of ß-actin in motor neurons.

### ß-actin is not required for motor neuron viability

**Figure 2:**
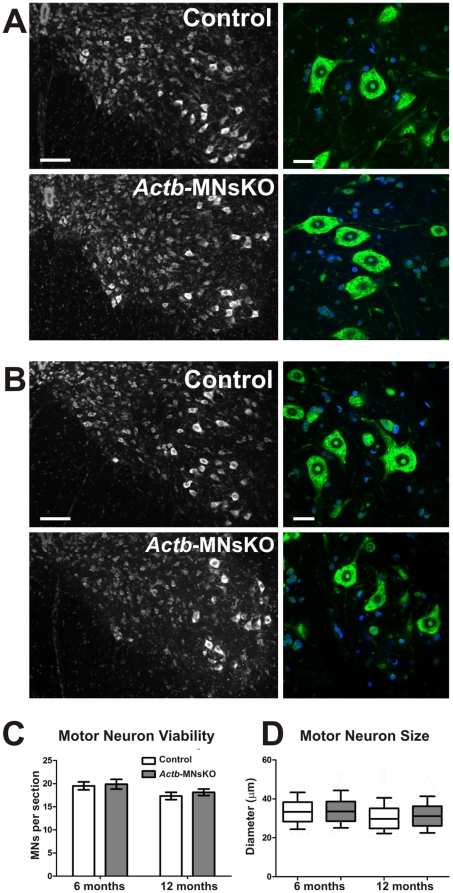
ß-actin is not required for motor neuron viability *in vivo*. (**A**) Representative images of Nissl stained spinal cord sections from the lumber enlargement of 6 and (**B**) 12 month old mice. Magnified images at right show no indication of chromatolysis in motor neurons. DAPI staining is in blue. Scale bar 100 µm left, 30 µm right. (**C**) Quantification of motor neurons per section at 6 and 12 months of age (*n* = 3 for each genotype per time point). No statistically significant differences were observed between control and *Actb*-MNsKOs at either time point. (**D**) Quantification of motor neuron size at 6 and 12 months of age. Data plotted as mean ± standard error of the mean.

Motor neuron viability in *Actb*-MNsKO mice was assessed by quantifying Nissl stained motor neurons in the ventral horns of spinal cord sections. At 6 and 12 months of age, no significant differences were observed in the number of motor neurons per section in *Actb*-MNsKO compared to controls, indicating that ß-actin is not required for motor neuron viability *in vivo* (Figure 2A–C). Motor neuron size was also examined to quantitatively assess cell structure in motor neurons deficient for ß-actin. At both 6 and 12 months of age, motor neuron diameter was comparable between control and *Actb*-MNsKO mice (Figure 2D). Although we did observe a significant difference (*p* = 0.03) in motor neuron diameter at 12 months of age, the difference of less than 1 µm is likely not biologically relevant.

### NMJ maturation and maintenance in *Actb*-MNsKO mice

We next examined motor axon patterning and maintenance in *Actb*-MNsKO mice. Motor axon transit to target muscles occurs between E9 and E11–12, although NMJs are not fully mature until approximately two weeks after birth [38], [39]. Because of the rapid outgrowth of axons shortly after motor neuron birth and Mnx1-Cre expression, we were not able to conclude that ß-actin protein was completely eliminated from motor neurons prior to motor axons reaching their final targets. However, we were interested in examining motor axon morphology and positioning following DNA recombination of the *Actb* locus, and thus the role of new ß-actin transcripts in late motor axon patterning. Given that recombination of the *Actb* locus occurs shortly after Mnx1-Cre expression at E12.5 (Figure 1D), synthesis of ß-actin from mRNA transcribed after E12.5 cannot occur.

**Figure 3:**
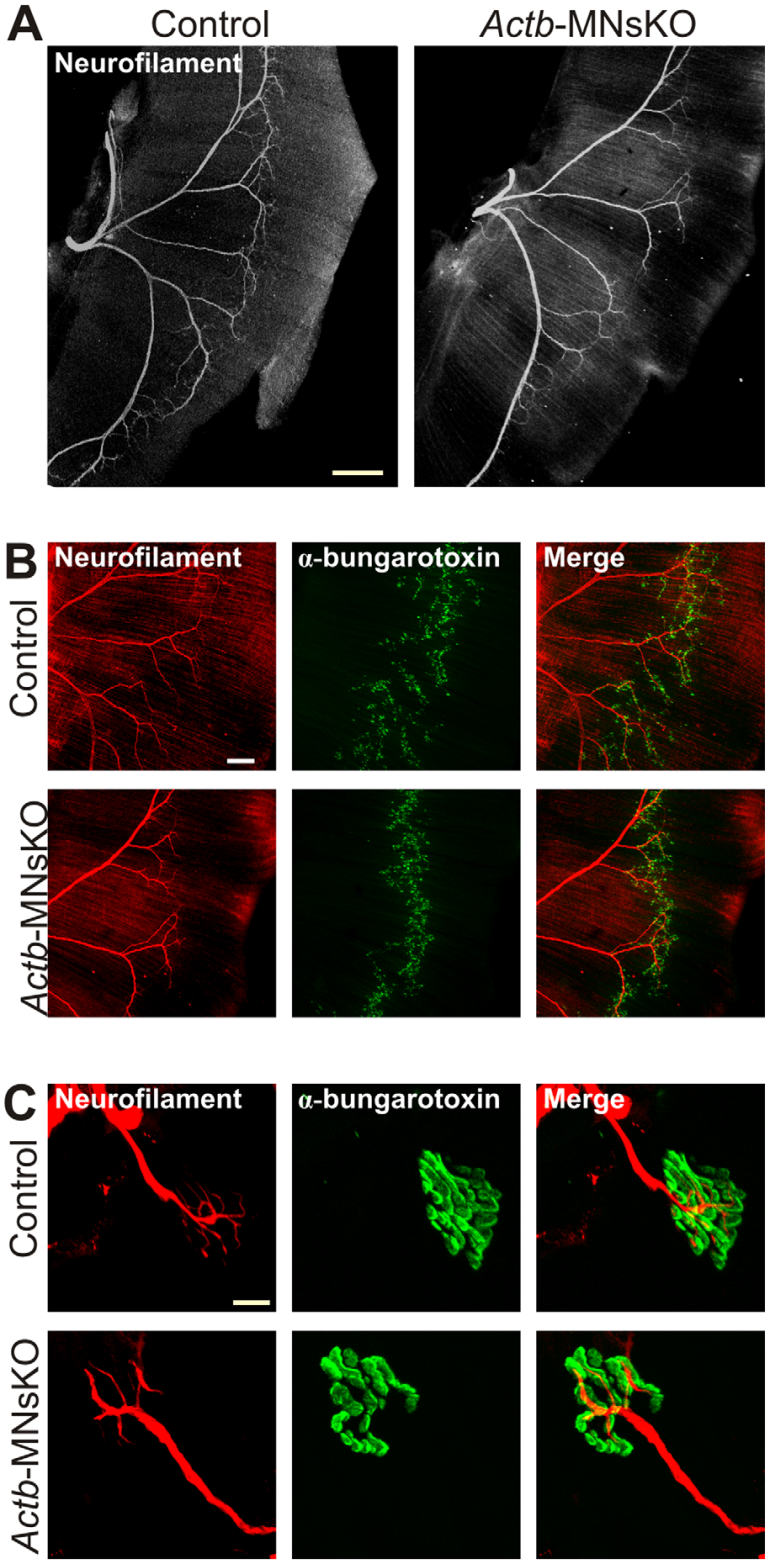
Neuromuscular junction development and morphology in *Actb*-MNsKO mice. (**A**) E16.5 left diaphragms stained with an anti-neurofilament antibody to label motor axons. Scale bar 300 µm. (**B**) Magnified images of E16.5 diaphragms stained with anti-neurofilament antibodies and Alexa 488 conju gated α-bungarotoxin. No indication of excessive branching or axons extending beyond the motor endplate was observed. Scale bar 150 µm. (**C**) Neurofilament and α-bungarotoxin staining of NMJs in the diaphragms of adult mice with no morphological defects observed. Scale bar 10 µm.

We used the developing mouse diaphragm as a model for assessing axonal guidance, where motor axons first reach the diaphragm between E11–12, but continue to branch and respond to guidance cues up to E15, when the mature innervation pattern is reached [40], [41]. Whole-mount E16.5 diaphragms from control and *Actb*-MNsKO embryos were stained with antibodies against neurofilament to label motor axons and fluorescently conjugated α-bungarotoxin to mark acetylcholine receptors at the motor end plate. Previous studies have shown defects in motor axon guidance can result in excessive motor axon branching or axons that fail to halt at the motor endplate [42]–[44]. We observed no indication of excessive motor axon branching (Figure 3A) or axons that extended beyond the motor end plate (Figure 3B). These results indicate that translation of ß-actin mRNA transcribed after E12.5 is not required for the late stages of motor axon guidance and elongation in the diaphragm.

Actin is also hypothesized to be an important component of the pre-synapse at mature NMJs, with roles in synaptic vesicle organization and endocytosis [45]–[48]. Using anti-neurofilament antibodies and α-bungarotoxin to label motor axons and acetylcholine receptors respectively, we stained whole-mount diaphragms from 4 month old control and *Actb*-MNsKO mice to assess NMJ stability and morphology. Nerve terminals were highly branched and overlapped extensively with acetylcholine receptors (Figure 3C), with no indication of retraction bulbs or neurofilament aggregation as seen in SMA mouse models [17], [18], [20], [21]. Post-synaptic acetylcholine receptors exhibited a highly folded morphology consistent with normal motor endplates (Figure 3C). Thus, the observation of mature motor endplates together with morphologically normal nerve terminals indicates that ß-actin is not required for the stability or long-term maintenance of NMJs.

### *In vivo* muscle function of *Actb*-MNsKO mice

**Figure 4:**
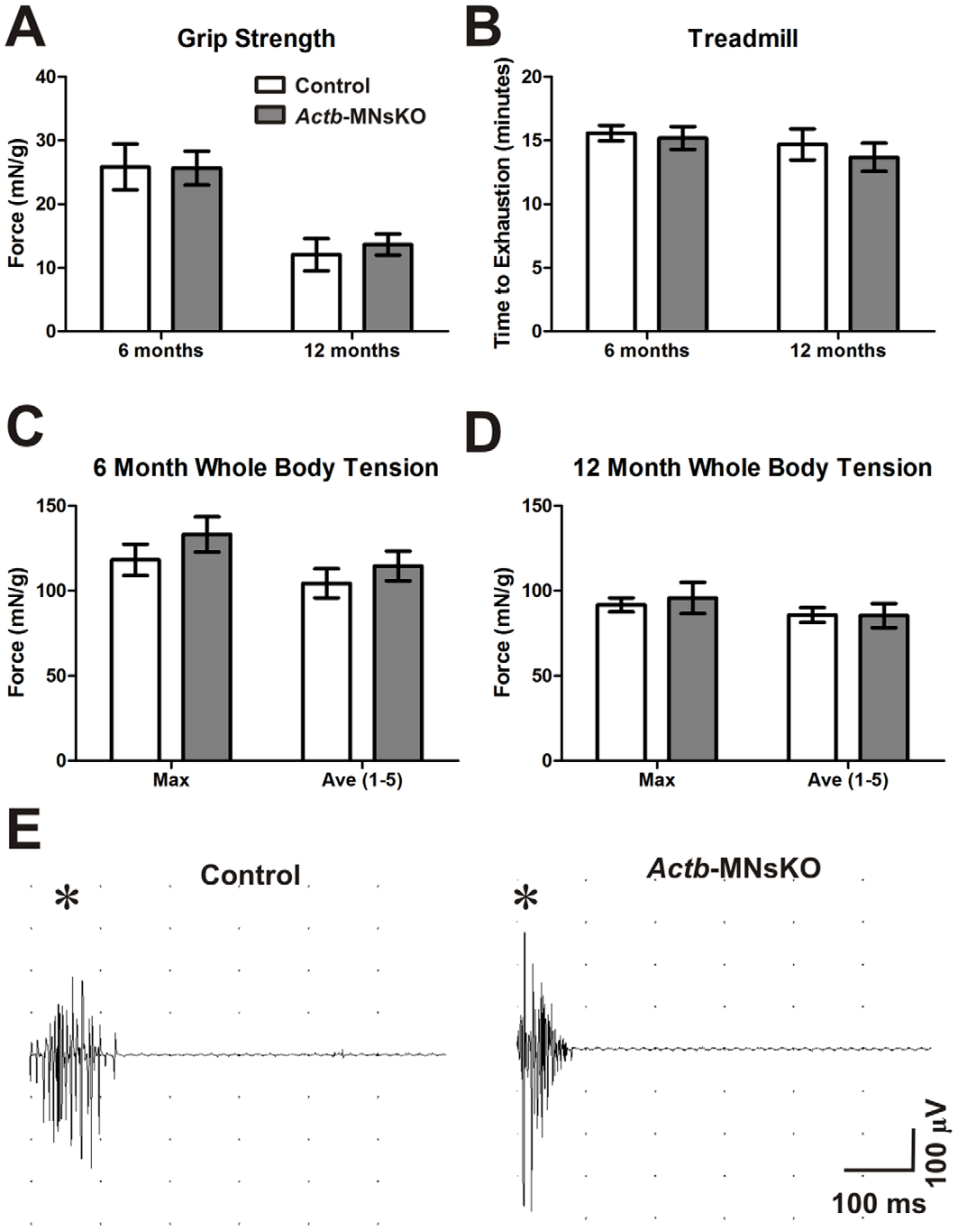
*In vivo* muscle function of *Actb*-MNsKO mice. (**A**) Grip strength, (**B**) treadmill, and (**C**) whole-body tension analysis performed on 6 and 12 month old control and *Actb*-MNsKO mice. *n*≥3 animals per genotype per time point for all experiments. No significant differences were observed between control and *Actb*-MNsKO mice in any assay at either time point. Error bars represent standard error of the mean. (**D**) Electromyography of 6 month old control and *Actb*-MNsKO mice showing no indication of fibrillation or fasciculation (*n* = 3 mice per genotype). Asterisks indicate depolarization due to initial needle insertion.

Although morphologically normal, subtle defects in pre-synaptic organization not visible by light microscopy could preclude normal NMJ synaptic transmission. In order to assess NMJ function in *Actb*-MNsKO mice, we used three physiological assessments of motor neuron function *in vivo*. Control and *Actb*-MNsKO mice at 6 and 12 months of age were subjected to grip strength, treadmill, and whole-body tension analyses to assess muscle performance and endurance. No significant differences were observed at either time point in any of the assays, suggesting that NMJ physiological function was not impaired in *Actb*-MNsKO mice (Figure 4A–D). CNS-*Actb*KO mice also presented with unimpaired motor function (Figure S1B–C) comparable to the *Actb*-MNsKO mice, thus providing independent confirmation of our findings in the *Actb*-MNsKO line (Figure 4A–D). Because a mild mouse model of SMA presented with EMG abnormalities [49], we also examined 6 month old control and *Actb*-MNsKO mice for spontaneous muscle depolarization consistent with motor neuron degeneration. No fibrillations or fasciculations were detected in *Actb*-MNsKO mice, further confirming the functional integrity of NMJs with ß-actin deficient motor neurons.

### *Actb*-MNsKO skeletal muscle does not show indications of atrophy or denervation

**Figure 5:**
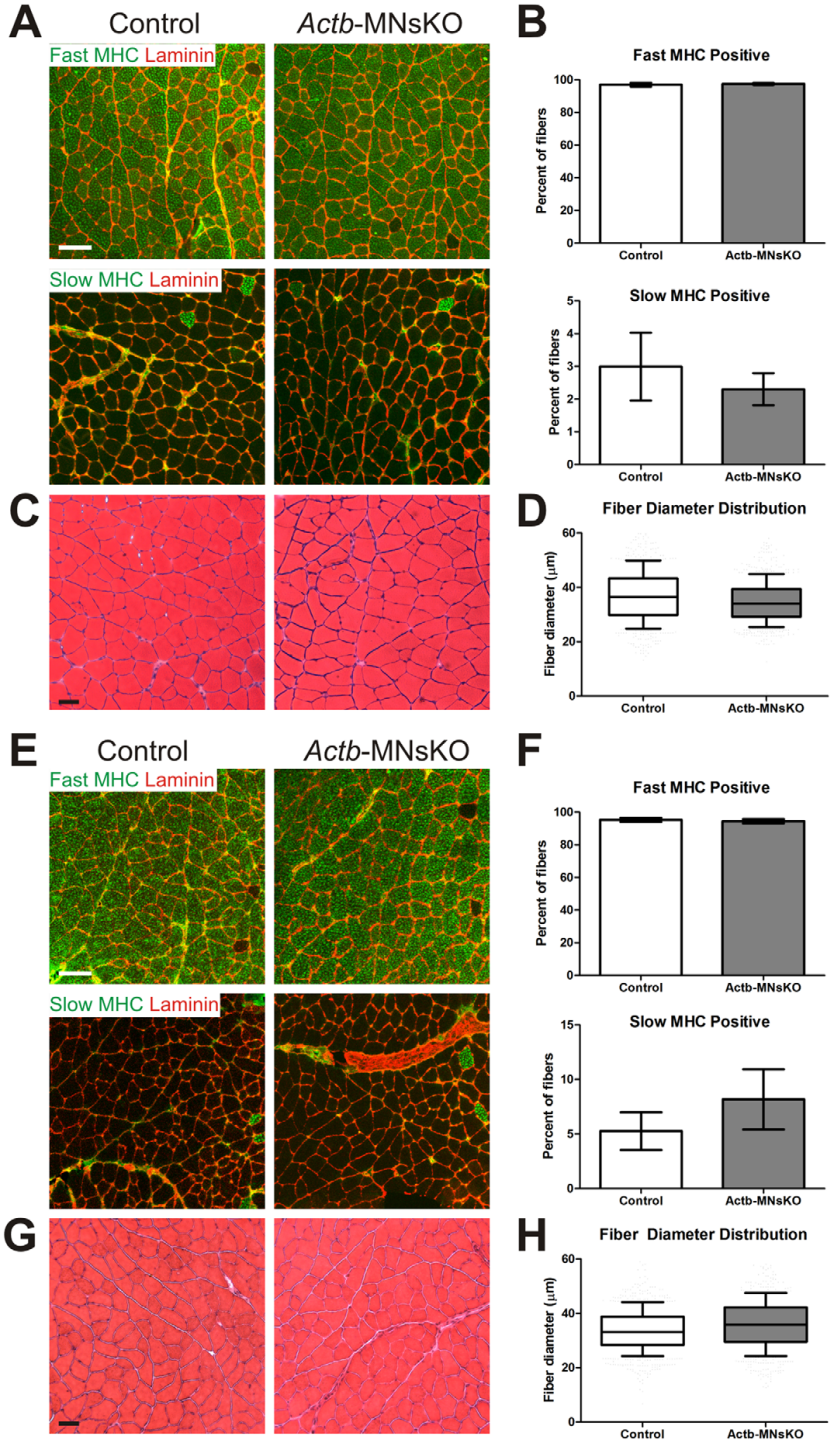
Histological analysis of skeletal muscle in *Actb*-MNsKO mice. Fiber type composition and distribution was assessed in the gastrocnemius muscle of 6 and (**A–B**) and 12 month old (**E–F**) control and *Actb*-MNsKO mice. Antibodies to fast and slow myosin heavy chain were used to identify fast and slow twitch fibers respectively. Anti-laminin staining was used to delineate the borders of muscle fibers. Scale bar 100 µm. Fiber diameter was analyzed from hematoxylin and eosin stained sections of the gastrocnemius muscle at 6 (**C**) and 12 months (**G**) of age. Scale bar 50 µm. Box and whisker plots of fiber diameter distribution at 6 (**D**) and 12 (**H**) months of age.

We next examined the histological properties of skeletal muscle from *Actb*-MNsKO mice for subtle or early signs of denervation. In a number of motor neuron diseases, selective loss of a particular fiber type or fiber type clustering can occur and is consistent with early motor neuron degeneration [50]–[52]. We stained gastrocnemius muscle sections from control and *Actb*-MNsKO mice at 6 and 12 months of age with antibodies to fast and slow myosin heavy chain to label fast and slow twitch muscle fibers respectively. No indication of fiber type clustering was observed in *Actb*-MNsKO mice and percent fiber type composition of the gastrocnemius up to 12 months of age was comparable to controls (Figure 5 A–B, E–F). Muscle fiber diameter was analyzed to determine whether any indication of muscle atrophy was present. Although significant differences were observed in fiber diameter between controls and *Actb*-MNsKO mice at both 6 and 12 months (Figure 5C–D, G–H), the small differences of approximately 2 µm are unlikely to be of physiological relevance, consistent with no significant differences observed in muscle performance at either 6 or 12 months in *Actb*-MNsKO mice (Figure 4A–D). Thus, ß-actin is not essential for the long-term stability and function of motor neurons and NMJs as revealed by histological analysis of skeletal muscle.

### ß-actin is not required for functional motor axon regeneration

Because ß-actin protein is estimated to have a half-life of 2–3 days [53], it remains possible that the normal motor axon outgrowth observed in *Actb*-MNsKO embryos was due to persistence of ß-actin expressed prior to gene recombination. To address the role of ß-actin in axonal elongation more directly, we used a tibial nerve crush model to examine motor axon regeneration in mice at 12 weeks of age, long after recombination of the *Actb* locus between E9.5 and 12.5 [24] (and Figure 1E).

**Figure 6:**
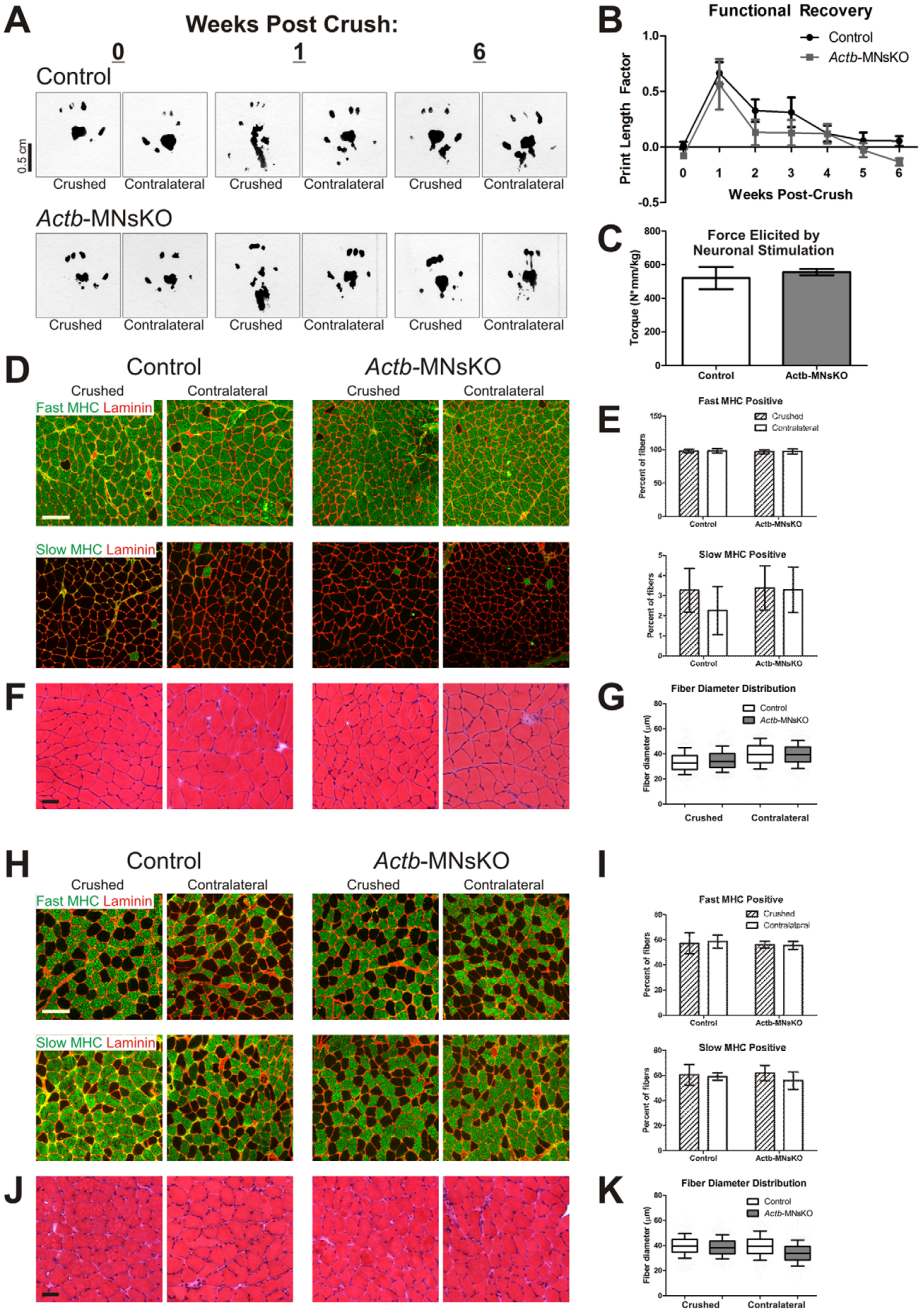
Peripheral nerve regeneration is not impaired in *Actb*-MNsKO mice. (**A**) Representative paw prints from the crushed and contralateral side of control and *Actb*-MNsKO mice at 0, 1 and 6 weeks post-crush. (**B**) Quantification of PLF functional recovery (*n* = 4 mice per genotype). No significant differences were observed between control and *Actb*-MNsKO mice up to 5 weeks post-crush. (**C**) Force elicited by neuronal stimulation did not differ in *Actb*-MNsKO as compared to controls, indicating that axonal function had been restored (*n* = 3 mice per genotype). Fiber type composition and distribution in the crushed and contralateral side of the gastrocnemius (**D–E**) and soleus (**H–I**) muscle at 6 weeks post-crush. Scale bars 100 µm. Fiber diameter distribution in the crushed and contralateral side of the gastrocnemius (**F–G**) and soleus (**J–K**) muscles at 6 weeks post-crush. Scale bar 50 µm. Three mice per genotype were analyzed for muscle histology.

Analysis of the print length factor (PLF) was used as a real-time readout for functional recovery and has been previously validated in mice [54]–[56]. Before nerve crush, the PLF of control and *Actb*-MNsKO mice did not significantly differ from each other and approached zero, consistent with an uninjured, normal PLF (Figure 6A and B). One week after tibial nerve crush, both control and *Actb*-MNsKO mice demonstrated a significant increase in PLF compared to pre-injury values (*p*<0.05), but did not significantly differ from each other, indicating that the level of injury was functionally equivalent between genotypes (Figure 6A–B). Both control and *Actb*-MNsKO mice followed a similar trend in functional recovery and did not significantly differ from each other up to 5 weeks post-crush (Figure 6A–B).

To complement the PLF recovery analysis, we also assessed motor neuron regeneration and reinnervation via nerve stimulation and analysis of muscle performance *in vivo*. At 6 weeks post-crush, the left (crushed-side) paw of sedated mice was placed on a lever to measure torque generated by neuronal activation of the gastrocnemius/soleus complex. Both control and *Actb*-MNsKO mice generated equivalent amounts of maximal plantarflexion torque, providing further evidence that ß-actin is not required for functional reinnervation of skeletal muscle following nerve crush (Figure 6C).

Finally, muscle histology was analyzed as in Figure 5 to determine whether any subtle indications of reinnervation defects were present. Fiber type composition did not significantly differ between the crushed side gastrocnemius of control and *Actb*-MNsKO, and neither significantly differed from their respective contralateral side, further indicating functional innervation was restored (Figure 6D–E). The same was true for the soleus, where fiber type composition was comparable between the crushed and contralateral sides with no indication of significant fiber type grouping (Figure 6H–I). 

Fiber diameter analysis showed a moderate decrease in fiber diameter of the crushed side gastrocnemius in both control and *Actb*-MNsKO mice as compared to the contralateral side, indicative of temporary denervation and subsequent reinnervation following motor axon regeneration (Figure 6G). Although fiber diameter significantly differed between the crushed side gastrocnemius of control and *Actb*-MNsKO mice, the difference of 1.29 µm is not likely biologically relevant. Muscle fiber atrophy due to denervation was less pronounced in the soleus, with no significant difference in fiber diameter between the crushed and contralateral side of control solei muscles (Figure 6K). For reasons that are not clear, the contralateral soleus of *Actb*-MNsKO mice deviated significantly from that of the control contralateral soleus, although we would expect a larger decrease in fiber diameter if atrophy was occurring due to muscle denervation. Additionally, fiber diameter of the crushed side and fiber type composition and distribution of both sides did not differ between controls and *Actb*-MNsKO mice (Figure 6H–K), further suggesting that this observed difference is not likely due to aberrant reinnervation.

## Discussion

Here we report the first *in vivo* characterization of ß-actin function in motor neurons using a motor neuron specific ß-actin knock-out mouse. ß-actin deficient motor neurons were viable and formed morphologically and functionally normal NMJs based on immunofluorescence and *in vivo* muscle performance assays. Skeletal muscle histology supported these findings and revealed no indication of denervation up to 12 months of age. Finally, motor axon regeneration proceeded normally in the absence of ß-actin, which altogether suggests that ß-actin plays a non-essential role in mature motor neurons *in vivo*.

The dispensability of ß-actin in motor neurons *in vivo* was surprising given that deficits in ß-actin mRNA and protein localization correlated strongly with growth cone and axon elongation defects observed in cultured SMA motor neurons [12]. However, a number of additional studies on SMA mouse models have not identified similar defects *in vivo*, suggesting that SMA is not likely caused by aberrant motor axon elongation or guidance [17]–[19]. Instead, defects in NMJ maturation were reported in these SMA mouse models, with abnormalities in pre-synaptic architecture and delayed maturation of the post-synaptic motor endplate [17], [18], [20]–[22]. The finding that voltage gated calcium channel delocalization correlated with the misregulation of ß-actin in cultured SMA motor neurons suggests that an improperly formed pre-synaptic actin cytoskeleton could contribute to synaptic transmission defects, thus hindering NMJ maturation in SMA mice [13]. However, we have shown that NMJ maturation proceeds normally in *Actb*-MNsKO mice (Figure 3C), indicating that the NMJ maturation defects observed in SMA mice are not likely due to a loss-of-function of ß-actin in the nerve terminal. Yet, we cannot rule out the possibility that the mislocalization of ß-actin contributes to SMA pathogenesis by some other mechanism, perhaps by disrupting the subcellular localization of other proteins or that some compensatory mechanism occurring in *Actb*-MNsKO mice does not also occur in SMA mouse models.

While studies in SMA mice have not revealed axon guidance or elongation defects *in vivo*, the hypothesis that the localization and local translation of ß-actin is important for normal motor neuron development cannot be ruled out. Although the timing of Cre expression limited our ability to assess early motor axon guidance and elongation in *Actb*-MNsKO mice, analysis of axonal regeneration following nerve crush allowed us to study axonal elongation in a system that recapitulates many properties of axonal development. Additionally, previous studies suggested that ß-actin has an important role in peripheral nerve regeneration with reports indicating that ß-actin expression is upregulated in response to sciatic nerve crush *in vivo*, and that ß-actin localization is required for growth cone formation and axon elongation in injury-conditioned dorsal root ganglion cultures [57], [58]. However, ß-actin deficient motor neurons had no functional defects or delays in axonal regeneration following nerve crush *in vivo* (Figure 6), suggesting that other actin isoforms or components of the cytoskeleton such as microtubules are likely sufficient for functional axonal regeneration and motor neuron function *in vivo*.

The dispensability of ß-actin in mature motor neurons is also surprising given that loss of ß-actin in hair cells of the inner ear and skeletal muscle results in mice with distinct phenotypes [23], [71]. Thus, the requirement for ß-actin *in vivo* likely varies in a tissue dependent fashion. Given the enormous diversity of neurons in the central nervous system, it remains possible that expression of ß-actin may only be required by some neurons and not others. Additional studies using Cre lines expressed by distinct populations of neurons besides motor neurons will be required to determine the relative importance of ß-actin to neuronal function *in vivo*.

## Materials and Methods

### Ethics Statement

The experimental protocols in this study were reviewed and approved by the University of Minnesota Institutional Animal Care and Use Committee (IACUC) and approved on August 13^th^, 2010 (IACUC Protocol #0907A69551).

### Mouse Lines

Motor neuron specific ß-actin knock-out mice (*Actb*-MNsKO) were generated by crossing *Actb^flox/flox^* [23] mice with the Mnx1 (also known as HB9, Hlxb9) – Cre line (Stock# 006600, Jackson Labs, Bar Harbor, ME) to generate mice homozygous for the *Actb* floxed allele and hemizygous for the Mnx1-Cre allele. Mice homozygous for the *Actb* floxed allele but lacking Cre were used for controls (*Actb^flox/flox^*). Mnx1-Cre expression in all or nearly all motor neurons has been previously verified by crossing to ROSA26 reporter strains [28]–[30], [59] and used to target Cre recombinase expression in gene knock-out or motor neuron ablation studies starting at embryonic day 9.25–9.5 (E9.25–E9.5) [24], [27]–[32], [60]–[62]. CNS-*Actb*KO mice were generated in a similar fashion to the *Actb*-MNsKO mice but with the Nestin-Cre line instead (Stock# 003771, Jackson Labs, Bar Harbor, ME). Nestin-Cre is expressed throughout the nervous system with Cre expression beginning at E10.5 [35], [36]. Genotyping for the *Actb* allele and Cre transgene were performed as described [23], [63]. For timed matings, the morning a vaginal plug was found was designated E0.5.

### ß-galactosidase Staining

Mnx1-Cre expression and activity were confirmed by crossing control and *Actb*-MNsKO mice to a ROSA26 reporter line (Stock# 003474, Jackson Labs, Bar Harbor, ME). Staining of E12.5 embryos for ß-galactosidase activity was performed on whole mounts or cryosections as described previously [64]. Cross sections were counterstained with 0.1% Neutral red.

### Spinal Cord Immunofluorescence and Histology

Mice at 6 (6–8) and 12 (12–14) months of age were anesthetized and transcardially perfused with cold phosphate buffered saline (PBS) followed by 4% paraformaldehyde (PFA, Electron Microscopy Sciences, Hatfield, PA) in PBS. The spinal cord was isolated by laminectomy and post-fixed in 4% PFA for 2 hours at 4°C. Isolated spinal cords were then cryoprotected for 2–3 days in 30% sucrose in PBS at 4°C. The lumbar enlargement of spinal cords were finally frozen in liquid nitrogen cooled isopentane and embedded in OCT (TissueTek, Torrance, CA). For immunofluorescence, 20 µm cryosections were cut and post-fixed in 4% PFA for 10 minutes, washed briefly in PBS, and post-fixed in ice cold 100% methanol for 10 minutes. Fixed sections were then washed in wash buffer (PBS+0.3% Triton X-100 (Sigma, St. Louis, MO)) followed by blocking in blocking buffer (3% bovine serum albumin (BSA, Sigma) in PBS+0.3% Triton X-100) for 1 hour at room temperature. Goat anti-choline acetyltransferase (AB144P; Millipore, Billerica, MA, 1∶10) was incubated overnight in 1% BSA+0.3% Triton X-100 at 4°C, while FITC-conjugated mouse anti-ß-actin (ab6277; Abcam, Cambridge, MA, 1∶75)) was incubated with 2° antibody (anti-goat Alexa Fluor 568; Invitrogen, Carlsbad, CA, 1∶500) the following day for 1–2 hours at room temperature. Sections were washed in wash buffer and mounted in SlowFade Gold antifade reagent with DAPI (S36938; Invitrogen). Images of control, *Actb*-MNsKO, and CNS-*Actb*KO sections were obtained and processed under identical conditions at the Biomedical Image Processing Laboratory with an Olympus FluoView FV1000 laser scanning confocal microscope and processed equivalently with Adobe Photoshop.

Fluorescent Nissl staining was performed on 20 µm cryosections using a 1∶100 dilution of NeuroTrace (N21480; Invitrogen) following the manufacturer's instructions. At least 10 sections >150 µm apart were used for quantification of Nissl stained motor neurons that met the following criteria used previously [65]: 1) Located below a horizontal line drawn tangent to the bottom of the central canal, 2) visible and distinct nucleolus with robust, globular cytoplasmic staining, and 3) diameter at the largest point >20 µm. Motor neuron size was assessed on the same sections by measuring the diameter at the largest point of all motor neurons with a minimum diameter of >20 µm using ImagePro software. Three mice per genotype per time point were characterized.

### Whole Mount Muscle Immunostaining

E16.5 diaphragms were dissected in 1× PBS and fixed in 4% PFA in 0.1 M PBS (0.019 M monobasic sodium phosphate, 0.081 M dibasic sodium phosphate heptahydrate, pH 7.4) for 30 minutes at room temperature. Fixed muscles were briefly washed in 0.1 M PBS followed by incubation in 0.1 M glycine in 0.1 M PBS for 1 hour. Diaphragms were then blocked in blocking buffer (4% BSA+0.5% Triton X-100 in 0.1 M PBS) overnight at 4°C. Mouse anti-neurofilament 1° antibody (2H3; Developmental Studies Hybridoma Bank, Iowa City, IA, 1∶200) was incubated in blocking buffer overnight at 4°C. Diaphragms were then washed in wash buffer (0.1 M PBS+0.5% Triton X-100) and incubated with anti-mouse 2° antibodies conjugated to Alexa Fluor 568 (A11031; Invitrogen, 1∶200) and α-bungarotoxin conjugated to Alexa Fluor 488 (B13422; Invitrogen, 1∶200) overnight at 4°C. After washing in wash buffer, diaphragms were flat mounted on slides with SlowFade Gold antifade reagent (S36936; Invitrogen).

Adult (4 month old) diaphragms were stained as described [66] with slight modification. 1% PFA was used as a fixative while 2H3 antibody and fluorescent α-bungarotoxin were used as described above.

### *In vivo* Muscle Performance and EMG

*In vivo* muscle performance was assessed at 6 and 12 months of age (*n*≥3 mice per genotype per time point). Forelimb grip strength measurements were made using a computerized grip strength meter (Columbus Instruments, Columbus, OH) following standard protocols [67], [68]. For each subject at each time point, five trials were conducted with the top three averaged and normalized to body mass. Maximal exercise performance was assessed using a Columbus instruments treadmill as reported previously [69]. Whole-body tension analysis was performed as described previously [70]. Data are reported as the maximum pulling force generated (WBT_Max_), and the average of the top five pulls (WBT_Ave(1–5)_). Electromyography (EMG) recordings were made using a TECA Synergy EMG monitoring system (Viasys Healthcare, San Diego, CA) with a 27 gauge disposable needle electrode (Medtronic, Minneapolis, MN). Six month old *Actb*-MNsKO and control mice were anesthetized with ketamine (100 mg/kg body weight [BW]) and xylazine (10 mg/kg BW) and maintained on a heating pad. A subdermal ground was placed subcutaneously on the back. The recording electrode was inserted into the gastrocnemius muscle and spontaneous electrical activity was recorded for 20 seconds in at least three different positions within the muscle.

### Muscle Histology

Fiber typing was performed at 6 and 12 months on the gastrocnemius muscle as described previously (*n*≥3 mice per genotype per time point) [69]. Fiber typing of gastrocnemius and soleus muscles following nerve crush was performed on muscles harvested 6 weeks post-crush (*n* = 3 mice of each genotype). For all experiments, three non-overlapping images were acquired with an Olympus FluoView FV1000 laser scanning confocal microscope and quantified using ImagePro software. For assessment of fiber diameter, hematoxylin and eosin stained sections were prepared and analyzed as described previously [70] with minor modification. Sections were analyzed from at least three different mice of each genotype at both 6 and 12 months. Three non-overlapping images were captured with a Zeiss Axiovert 25 microscope and >1000 fibers were analyzed for each genotype in all experiments.

### Nerve Crush

Twelve week old control and *Actb*-MNsKO mice (*n* = 4 for each genotype) were first anesthetized with 0.2% isofluorane. Under aseptic conditions, a 15 mm incision was made distal and parallel to the femur of the left leg through the skin and biceps femoris muscle. The tibial nerve was located and dissected away from musculature and fat. Upon isolation, the tibial nerve was crushed with a fine tip hemostat (13020-12; Fine Science Tools, Foster City, CA) clamped to the first notch for exactly 30 seconds. After verification of crush under a dissecting microscope, the bicep femoris and skin were closed with 6-0 sterile silk suture. The left tibial nerve of all mice was crushed while the right served as an inter-animal control. Mice were administered buprenorphine (0.05 mg/kg BW) immediately and then daily for three days post-surgery.

### Print Length Factor

Functional recovery after tibial nerve crush was assessed via determination of the Print Length Factor (PLF) as described previously [55] with slight modification. The hind paws of mice were dipped in black India Ink and mice were allowed to walk down a corridor lined with white paper. Three trials at each time point were conducted per mouse, and four sets of prints from the same stride were measured and averaged for each time point.

### *In vivo* Muscle Torque Analysis

Maximal isometric torque of the plantarflexors (i.e., posterior muscles gastrocnemius, soleus, plantaris) was measured *in vivo* using a muscle-lever servomotor (model 300B-LR, Aurora Scientific, Aurora, Ontario,Canada). Mice were anesthetized with a cocktail of fentanyl citrate (10 mg/kg BW), droperidol (0.2 mg/kg BW), and diazepam (5 mg/kg BW). The left hindlimb was shaved, aseptically prepared, and each mouse was positioned on a heated platform with its left foot placed in a metal foot plate attached to the servomotor and knee clamped to maintain positioning throughout the experiment. Two platinum electrodes (model E2–12, Grass Technologies, West Warwick, RI, USA) were inserted subcutaneously on either side of the sciatic nerve. To ensure that sciatic nerve stimulation would not elicit anterior muscle contractions the peroneal nerve was cut. A plantar-flexion contraction was elicited by electrical stimulation of the sciatic nerve via a stimulator and stimulus unit (models S48 and SIU5, respectively, Grass Technologies, West Warwick, RI, USA). The parameters for stimulation were set at a 200 ms contraction duration consisting of 0.5 ms square-wave pulses at 250 Hz. The voltage was adjusted from 3.0 to 9.0 V until maximal isometric torque was achieved.

### Statistical Analysis

All data are presented as mean ± standard error of the mean and calculated with GraphPad Prism 5 software (GraphPad Software, Inc.). T-tests were conducted to determine statistical significance when only two groups were compared while one-way ANOVA was used for groups of three or more accompanied by a Tukey post hoc test or Bonferroni post test in the case of the regeneration fiber diameter analysis. A p-value of <0.05 was considered significant.

## Supporting Information

Figure S1 **Characterization of motor neuron function in CNS-** ***Actb*** **KO mice.** (**A**) Ventral horns from cross sections of the lumbar enlargement of the spinal cord from adult control and CNS-*Actb*KO mice stained with a ß-actin specific antibody and DAPI to label nuclei. Arrows indicate motor neuron cell bodies. Scale bar 30 µm. (**B–C**) Eight-10 month old CNS-*Actb*KO mice do not present with deficits in motor function compared to controls as determined by grip strength (**B**) and whole-body tension assays (**C**). *n*≥4 mice per genotype. Data plotted as mean ± standard error of the mean.
(TIF)
