# Supplementary figures and images for: Axonal Regeneration and Neuronal Function Are Preserved in Motor Neurons Lacking ß-Actin In Vivo
Source: PLoS One. 2011 Mar 22;6(3):e17768. doi: 10.1371/journal.pone.0017768 (PMC3062555; doi:10.1371/journal.pone.0017768)

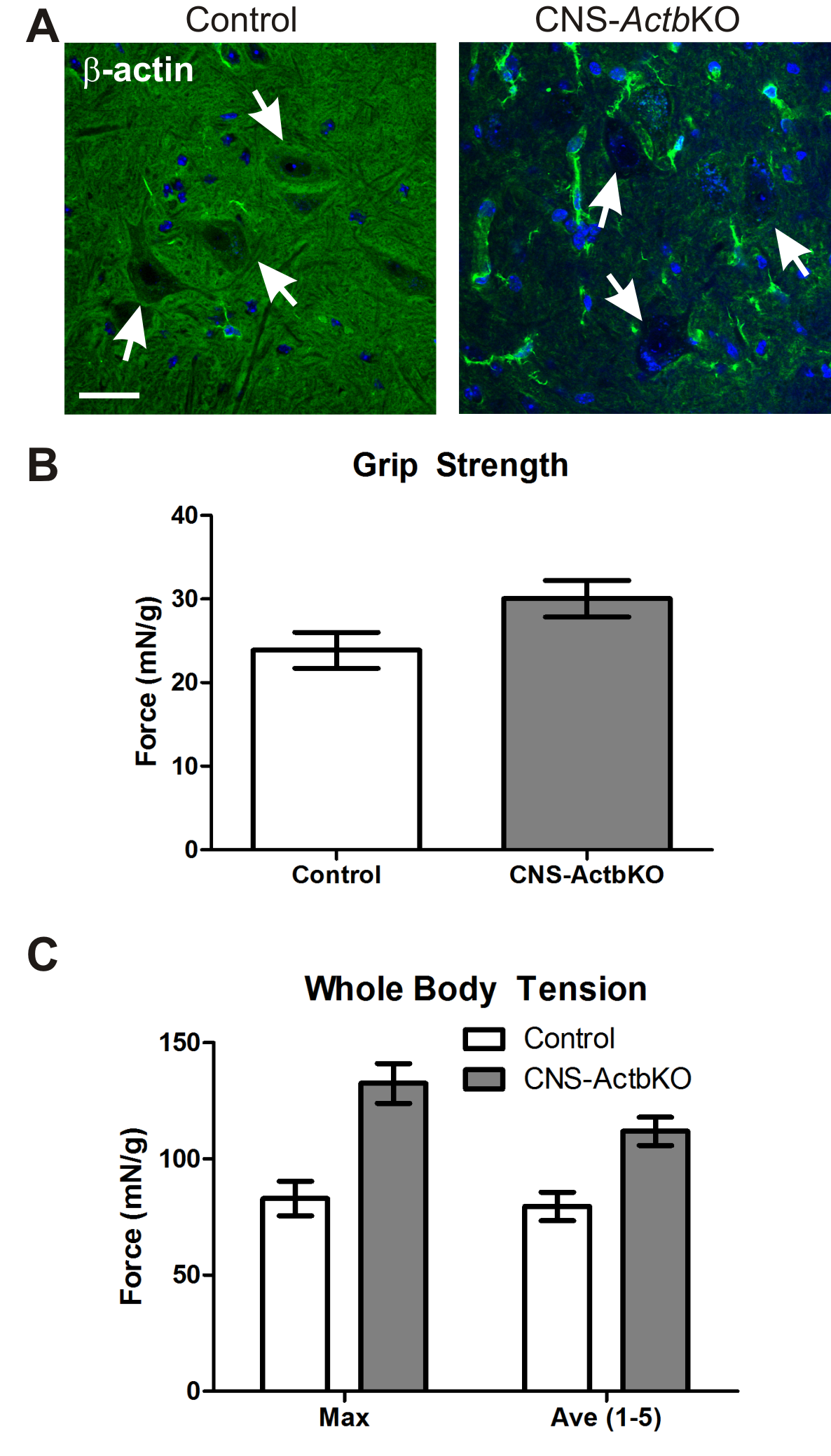

Supplement: Figure S1 — Characterization of motor neuron function in CNS- Actb KO mice. (A) Ventral horns from cross sections of the lumbar enlargement of the spinal cord from adult control and CNS-ActbKO mice stained with a ß-actin specific antibody and DAPI to label nuclei. Arrows indicate motor neuron cell bodies. Scale bar 30 µm. (B–C) Eight-10 month old CNS-ActbKO mice do not present with deficits in motor function compared to controls as determined by grip strength (B) and whole-body tension assays (C). n≥4 mice per genotype. Data plotted as mean ± standard error of the mean. (TIF) [file pone.0017768.s001.tif]
